# Changes in emission regime for nitrogen and sulfur in Germany and its impact on a spruce forest measured over a period of 35 years

**DOI:** 10.1002/jeq2.70147

**Published:** 2026-01-29

**Authors:** A. Göttlein, W. Weis, S. Raspe

**Affiliations:** ^1^ Professorship of Forest Nutrition and Water Resources TU München Freising Germany; ^2^ Bavarian State Institute of Forestry Freising Germany

## Abstract

In Germany during several decades, emissions and thus the chemical climate affecting forests have changed significantly. The effects of these changes on the element balance of forests can be documented only by long‐term observations, as has been done at the Höglwald site (Southern Bavaria) since 1985. Since then, structural changes in agriculture have led to a reduction in emissions of reduced nitrogen (NH_3_). There was also a slight decrease in emissions of oxidized nitrogen (NO_x_). Air pollution control measures, especially in the 1980s, led to a particularly drastic reduction of sulfur emissions (SO_2_). Consequently, inputs to the ecosystem decreased by almost 95% between 1985 and 2020. Dry deposition nowadays plays practically no role for this element. High nitrogen inputs, dominated by reduced nitrogen, have led to a high proton production through N transformations. This has gradually reduced the buffering capacity of the topsoil. Comparing measured fluxes shows that with decreasing sulfur inputs, the sulfur stored in the topsoil from times of high deposition was remobilized. At the Höglwald, this process occurred rather clearly over a period of about 28 years and has resulted in only about 11% of the initial amount of sulfur being still present in the topsoil (humus layer + mineral soil down to 40 cm) in 2020. Forestry should take the changed chemical conditions into account in its nutrient management.

## Core Ideas

- Changes in the emission regime should be detectable in the input into a forest ecosystem.
- Changes in input result in changes in soil–chemical properties and in changes in output by seepage.
- There may be a delay between changes in input and changes in output.
- This study demonstrates the importance and value of long‐term forest ecosystem monitoring.

## Plain Language Summary

Air pollutants emitted from agriculture, industry, and transportation (NH_3_, NO_x_, SO_2_) affect natural ecosystems. In a spruce forest (Höglwald) in southern Bavaria, Germany, the input and output of nitrogen and sulfur were studied over 35 years (1985–2020). The study revealed that sulfur emissions, and thus sulfur inputs into the forest, decreased significantly due to successful air pollution control measures. The sulfur reserves stored in the soil during periods of high sulfur emissions were remobilized and discharged with the seepage water over a period of approximately 30 years. Despite a slight decrease in nitrogen deposition, the proton turnover associated with nitrogen conversion still leads to high soil acidification. The consequences of air pollution on forestry can only be documented and assessed through long‐term measurements.

**Abbreviations**
ICP Forests: International Cooperative Programme Forests
LWF: Bayerische Landesanstalt für Wald und Forstwirtschaft (Bavarian State Institute of Forestry)
StMELF: Bayerisches Staatsministerium für Ernährung, Landwirtschaft und Forsten (Bavarian State Ministry of Food, Agriculture and Forestry)

## 1 INTRODUCTION

Similar to the change in physical climate, the change in chemical climate is largely caused by human activity. The emissions from industry, agriculture, transport, and settlements release not only the greenhouse gas CO_2_, causing global warming, but also relevant quantities of the gases SO_2_, NH_3_, NO, and NO_2_, the latter two summarized as NO_x_ (European Environmental Agency, 2023). These emissions cause immissions that have a negative impact on terrestrial ecosystems, particularly forests (Stevens et al., 2020). A prominent example, the phenomenon of “acid rain,” was the subject of intensive research in the 1980s (Adriano & Havas, 1989; Berdén et al., 1987). The acidification of forest soils was considered a major cause of forest damage in the 1970s and 1980s (Ulrich, 1983, 1986, 1990; van Breemen, 1985). Additionally, the long‐lasting input of nitrogen compounds has led to a gradual but clear and visible change to a less species‐rich and more nitrophilous ground vegetation, already decades ago (Bürger, 1988; Eichhorn, 1995). This change even impacts nitrogen‐fixing plants (Moreno‐Garcia et al., 2024). To detect and evaluate the effects of chemical climate change on the element balance of a forest ecosystem, long time series of measurements are required. One of the longest time series in Central Europe, continuously running since spring 1984, is maintained at the Höglwald site, located in Bavaria between Augsburg and Munich. Measurements started at the time, when the International Cooperative Programme Forests (ICP Forests) was established within the framework of the United Nations Economic Commission for Europe. Within ICP Forests, in 1994, permanent intensive monitoring plots (Level II plots) were established (Thünen Institut, 2016). The Höglwald was integrated to the German Level II program and is now one of 19 spruce‐dominated German Level II plots. Together with the plots at Solling and Lange Bramke, it has a time series that began many years before the official start of Level II (Thünen Institut, 2022). The objective of our present work was to present, analyze, and discuss the development of the inputs of sulfur and nitrogen compounds from the atmosphere, as well as their respective discharges with seepage water over a period of 35 years (1985–2020). This allowed us to assess the response of a mature Norway spruce stand to ongoing, but also changing (in terms of quantity and quality) air pollution.

## 2 MATERIALS AND METHODS

### 2.1 Forest stand

The Höglwald forest (latitude 48.29023, longitude 11.07283) is located in the Tertiary Hills about 16 km south‐east of Augsburg and 50 km north‐west of Munich at an altitude of 540 m above sea level. The study stand originates from plantations and is the second generation of Norway spruce (*Picea abies*) after hardwood (Kreutzer & Bittersohl, 1986; Kreutzer & Weiss, 1998). The spruce stand exceeds the upper height class 40 of the spruce yield table according to Assmann and Franz (1963) and can therefore be classified in the uppermost yield level.

From autumn 1982 to spring 1984, six experimental plots were set up in the Höglwald forest to study the effects of acid irrigation and compensatory liming on a Norway spruce stand that was about 76‐year‐old in 1982 (Kreutzer & Bittersohl, 1986; Kreutzer & Weiss, 1998). The control plot of this investigation site has been in continuous operation since 1984. Because in 1984 data are not available for the entire year, the presentation of annual fluxes starts with the year 1985. In the early years, the measurement series was maintained as part of several university research projects. In 2010, the experimental site was transferred to the Bavarian Forest Ecosystem Monitoring Programme and has since been operated by the Bavarian State Institute of Forestry (LWF) as a Level II plot of the German and the international forest environmental monitoring (ICP Forests).

### 2.2 Climate

According to Walentowski et al. (2004), the Höglwald is located in the colline‐submontane to submontane altitudinal zone and has a suboceanic climate. The data from the nearest station of the Deutscher Wetterdienst (German Weather Service) in Augsburg (station number 00232; https://opendata.dwd.de) for the period 1985–2020 were used to characterize the climatic conditions during the time of observation. Mean annual temperature varied between 7.3°C and 10.0°C. A linear trend analysis revealed a statistically significant increase in temperature (*p* < 0.001) from 8.2°C to 9.4°C during the period 1985–2020. Annual precipitation values varied between 523 and 1054 mm with an average of 752 mm per year. There is no significant trend in precipitation over time. Even though the Höglwald is located about 70 km north of the Alps, their influence is recognizable there, as the climatic effect of the Alps with respect to precipitation in Bavaria still reaches this distance in the foreland (Bayerischer Klimaforschungsverbund, Meteorologisches Institut der Universität München, 1996). This is reflected in the fact that even in years with very low precipitation, the precipitation totals during the vegetation period (May–October) never fell below 300 mm. The Höglwald is therefore one of the climatically favored regions of Bavaria in terms of both temperature and precipitation.

### 2.3 Soil

The geological parent material consists of fine sediments of the Upper Miocene Freshwater Molasse with loess loam admixtures in the upper soil. A reference soil profile was analyzed at the beginning of the investigation period (Kreutzer & Bittersohl, 1986). The humus layer in the Höglwald was formed by a moder of about 5 cm thickness. The soil, a weakly podzolized Luvisol, had a low base saturation (<10%) down to 50 cm with pH(H_2_O) values (pH value of soil measured in distilled water) increasing with depth from 3.7 to 4.2. Below a depth of 1.3 m, significantly sandier layers followed, with pH(H_2_O) values of up to 5.3 and a base saturation of up to 78%. The latter is of great importance for the supply of calcium, magnesium, and potassium to the trees. The entire soil profile was free of stones, and only a few hydromorphic features were found in the subsoil. The soil thus can be deeply rooted and is characterized by a high water storage capacity.

### 2.4 Instrumentation and calculation of fluxes

Bulk precipitation was collected with three open polyethylene samplers on a meadow located about 1 km away from the study stand. Throughfall was sampled with 10 (until 2010) or 20 (from 2010) open samplers. Seepage water at a depth of 40 cm was collected with ceramic suction cups with 10‐fold replication up to 2010 and fivefold replication thereafter. The reduction in the number of suction cups was achieved by appropriate selection, so that means and ranges of the pre‐2010 time series were only marginally affected. Sampling soil water at 40‐cm depth provides a good estimate of the chemical quality of the water leaving the rooting zone, as the rooting of Norway spruce is very shallow in the area between the stems, and at the Höglwald, this area is dominating seepage (Pröbstle & Kreutzer, 1991).

**Core Ideas**

- Changes in the emission regime should be detectable in the input into a forest ecosystem.
- Changes in input result in changes in soil–chemical properties and in changes in output by seepage.
- There may be a delay between changes in input and changes in output.
- This study demonstrates the importance and value of long‐term forest ecosystem monitoring.

pH values were measured with a glass electrode in the unfiltered samples. Then, the water samples were filtered through 0.45‐µm membrane filters and stored at 4°C until analysis. Al, Mn, Fe, Ca, Mg, Na, and K were analyzed by Inductively Coupled Plasma Optical Emission Spectroscopy; Cl^−^, NO_3_^−^, and SO_4_^2−^ were determined by ion chromatography. At the beginning of the time series, NH_4_^+^ was measured using a colorimetric method (indophenol blue) and an autoanalyzer. From 2008 onward, NH_4_^+^ was also measured using ion chromatography. When the responsibility for sampling and analysis was transferred from the university to the state institute, the samples were measured in both laboratories on 19 sampling dates in parallel and the values compared. There was good agreement between the measurements with a slope of the comparative regression lines of the individual parameters between 0.88 and 1.10 (1.00 would be ideal).

Water fluxes were measured either directly (bulk precipitation and throughfall) or calculated using the mechanistic hydrological model LWF‐Brook90 (Federer, 2021; Hammel and Kennel, 2001; Weis et al., 2023). The modeled matrix potential and water content were compared with the corresponding measured values for quality control. The initial soil physical instrumentation of the site is described by Grimmeisen et al. (1986) and Pröbstle et al. (1991). Ion fluxes were calculated by multiplying the mean concentrations of each sampling date by the sum of the daily water fluxes for the respective sampling period.

### 2.5 Nitrogen and sulfur stock and uptake in the aboveground biomass

In November 2007, an intensive harvest of the aboveground biomass was carried out at the Höglwald site in order to quantify the nutrient storage of the stand. Five trees representative for the diameter in breast height distribution were felled and divided into the compartments: stem wood, stem bark, branches, twigs, and needles. The element contents determined in the laboratory were extrapolated to the stand using biomass functions. For further details, see Weis et al. (2009). The annual nutrient element incorporation into the individual compartments was estimated by calculating biomass production using the forest growth simulator SILVA 2.3 (Pretzsch et al., 2002) with the specific data of the stand (Manghabati et al., 2018).

## 3 RESULTS AND DISCUSSION

### 3.1 Development of nitrogen and sulfur emissions in Germany

**FIGURE 1:**
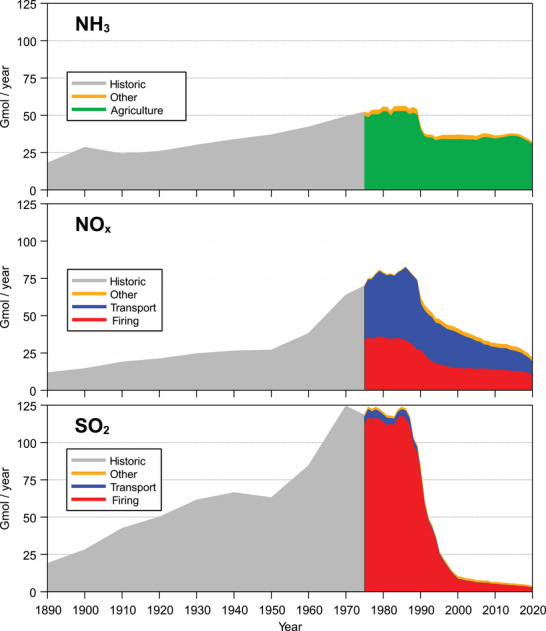
Development of emissions for NH_3_, NO_x_, and SO_2_ on the territory of the Federal Republic of Germany; the historical emission data for the period 1890–1975 originate from van Aardenne et al. (2001) for the OECD Europe region and were standardized to the first available value in 1975; for 1975–1989, the data of the former Western Germany (FRG) and the German Democratic Republic (GDR) were combined (Umweltbundesamt, 1993); from 1990 on, the data represent Germany as a whole (www.umweltbundesamt.de/daten); to allow direct comparability, all emission values were converted to a molar basis.

Emissions of reduced nitrogen showed an almost continuous increase from 1890 to 1988, with agriculture accounting for more than 90% of the emissions (Figure 1). With the reunification of the two German states in 1989 and the subsequent closure of most of the large livestock farms in the former German Democratic Republic, NH_3_ emissions decreased significantly. Since then, NH_3_ emissions have remained fairly constant at around 36 Gmol per year, with a declining trend from 2018 onward. Oxidized nitrogen showed a slight, continuous increase from 1890 to 1950. With the economic boom after World War II, NO_x_ emissions increased markedly from about 27 Gmol in 1950 to about 82 Gmol in 1986. The ratio of firing‐related to traffic‐related emissions was approximately 1:1.2. Reunification and the associated closure of many industrial emitters, as well as the mandatory installation of catalytic converters in motor vehicles starting in 1989 (Grieger, 2019), led to a significant reduction in NO_x_ emissions around 1990 in both the firing and the transport sector. After 1990, a continuous decrease in NO_x_ emissions could be observed, primarily due to the transport sector.

Sulfur emissions, more than 90% of which came from firing in the 1980s, showed the greatest change over time. During industrialization, SO_2_ emissions tripled from 1890 (about 20 Gmol) to 1950 (about 63 Gmol), with a slight dip during the years of World War II. From 1950 to 1970, SO_2_ emissions doubled again to about 124 Gmol, a level that was maintained until about 1987. The closure of large power plants on the territory of the former GDR after 1989, combined with the installation of flue gas desulfurization systems in the remaining power plants, led to a drastic reduction in SO_2_ emissions within a few years.

**FIGURE 2:**
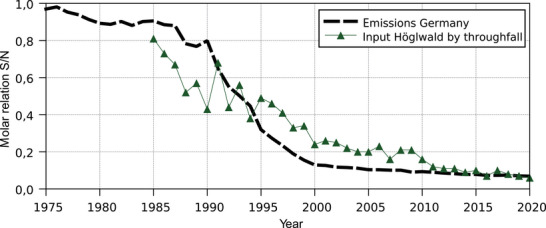
Molar sulfur/nitrogen (S/N) ratio of emissions in the territory of the Federal Republic of Germany in the years 1975–2020 and in throughfall at the Norway spruce forest Höglwald (Bavaria, Germany) for 1985–2020.

Over the period of the data series available from the German Environment Agency (since 1975), the reduction in sulfur emissions was much greater than the reduction of nitrogen emissions. This resulted in a significant shift in the molar ratio of the two elements, showing that not only the quantity (Figure 1) but also the overall chemical quality of total emissions changed considerably (Figure 2). While the S/N molar ratio was close to 1.0 in 1975, it fell to 0.07 in 2020, with a marked decrease occurring between 1990 and 2000.

### 3.2 Development of nitrogen and sulfur input at the Höglwald site

**FIGURE 3:**
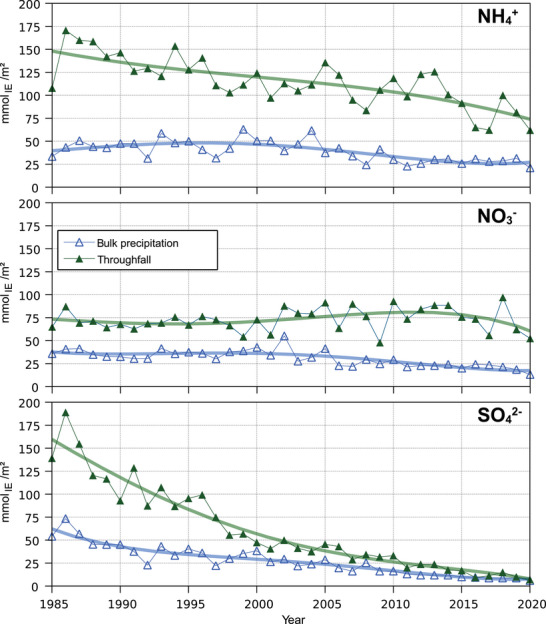
Development of the inputs of NH_4_^+^, NO_3_^−^, and SO_4_^2−^ with bulk precipitation and throughfall in the years 1985–2020 at the Norway spruce forest Höglwald (Bavaria, Germany); the polynomials shown (degree 3 and 4) serve to better visualize and interpret the development over time.

At the start of the monitoring period, N inputs with bulk precipitation were about 40 mmol_IE_ m^−2^ year^−1^ for both reduced (NH_4_^+^) and oxidized nitrogen (NO_3_^−^; Figure 3). By the end of the observation period in 2020, the input values for both forms of nitrogen had decreased slightly. This decrease was greater for oxidized nitrogen (to about 20 mmol_IE_ m^−2^ year^−1^) than for reduced nitrogen (to about 30 mmol_IE_ m^−2^ year^−1^). In contrast, in 1985, the input of reduced nitrogen into the soil by throughfall (about150 mmol_IE_ m^−2^ year^−1^) was more than twice as high as the input of oxidized nitrogen (about 70 mmol_IE_ m^−2^ year^−1^). Over time, a marked decrease in throughfall deposition of reduced nitrogen can be observed (to about 70 mmol_IE_ m^−2^ year^−1^), while only a slight decrease to about 60 mmol_IE_ m^−2^ year^−1^ in 2020 is indicated for oxidized nitrogen. For NH_4_^+^ in bulk precipitation as well as for NO_3_^−^ in throughfall, the difference between the beginning and end of the time series (polynomial interpolation) is lower than the 90% percentile of inter‐annual fluctuations. Thus, in these two cases, there is no interpretable trend within the time of observation. The resulting deposition of about 130 mmol_IE_ m^−2^ year^−1^ of total inorganic N in 2020 is still almost twice the net N accumulation in the aboveground biomass for 100‐year‐old Norway spruce trees at the Höglwald, which was estimated from our biomass investigation to be about 69 mmol_IE_ m^−2^ year^−1^.

Sulfur input in the form of sulfate decreased significantly in both bulk precipitation and throughfall. In 1985, the input by throughfall was about 160 mmol_IE_ m^−2^ year^−1^, or more than 2.5 times higher than bulk precipitation (about 60 mmol_IE_ m^−2^ year^−1^). The big difference between bulk precipitation (i.e., wet deposition to the forest canopy) and throughfall (i.e., wet deposition to the forest floor) is mainly caused by the deposition of gases, aerosols, and dust to the forest canopy (dry deposition), as the leaching of sulfate out of the canopy in natural systems is negligible (Adriaenssens et al., 2012). In 2020, both bulk precipitation and throughfall were in the order of about 10 mmol_IE_ m^−2^ year^−1^, indicating that the formerly dominating dry deposition of sulfate no longer plays a role. However, S deposition still was sufficient to meet the net S uptake by trees of about 5.5 mmol_IE_ m^−2^ year^−1^.

Calculating the molar S/N ratio in throughfall (Figure 2) reveals a decrease from about 0.8 in 1985 to about 0.07 in 2020. This decrease, both in its relative course and absolute values, fits very well with the course of the molar S/N ratio in the emissions of the Federal Republic of Germany. Although the decrease of the molar S/N ratio at the Höglwald is slightly less steep, Figure 2 highlights the link between changes in large‐scale emissions and inputs to the forest ecosystem.

### 3.3 Development of seepage output for nitrogen and sulfur

**FIGURE 4:**
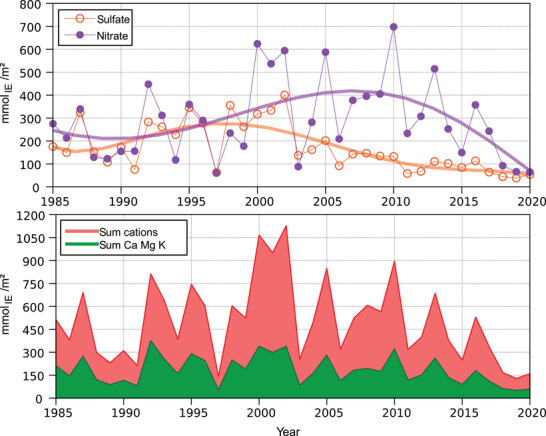
Development of NO_3_^−^, SO_4_^2−^, and cations in the leachate in a depth of 40 cm from 1985 to 2020 at the Norway spruce forest Höglwald (Bavaria, Germany); the polynomials shown (degree 5) serve to better visualize and interpret the development over time.

Discharges with seepage water (Figure 4) revealed much greater year‐to‐year variability than the inputs with bulk precipitation and throughfall (Figure 3). This is because a variety of processes in the soil (ion uptake by the plants, sorption and desorption, transformations of substances, etc.) have an influence on the ion concentration in the soil solution. Additionally, the amount of leachate is strongly influenced by the amount of precipitation and by plant transpiration. Particularly noticeable were the low output fluxes of the years 1997 (the year with the lowest annual precipitation) and 2003 and 2018–2020, years with severe summer drought in southern Germany (www.ufz.de/duerremonitor).

Looking at the polynomial trend lines in the case of nitrate discharge, an increase occurred during the period from 1991 to 2007, followed by a decrease until 2020. During the period of increased discharge, the NO_3_^−^ inputs with throughfall also were slightly increasing (see Figure 3) but cannot explain the magnitude of the observed increase in discharge. The sharp decrease in NO_3_^−^ discharge toward the end of the time series was likely due to the below‐average precipitation and seepage in the years 2018–2020 and should not be over‐interpreted. Sulfate discharge increased from 1987 to 1997 and steadily decreased thereafter. The simultaneous increase in nitrate and sulfate discharges at the beginning of the 1990s coincides with the significant rise in temperature during the observation period and may indicate an increase in mineralization.

For charge neutrality reasons, anions must be accompanied by cations when they leave the ecosystem with the leachate (Figure 4). Therefore, the inter‐annual pattern of the total cation discharge largely paralleled that of nitrate discharge, which was the dominant anion. At the Höglwald site, the dominant cation in terms of quantity in the leachate was aluminum (measured as Al, assumed as cation Al^3+^; also the other metals were measured as element and assumed as cations), accounting for an average of 42% of the total cation load over the observation period. As Al is not a nutrient, its discharge plays virtually no role in the nutritional status of the trees. However, the discharge of Ca, Mg, and K is of importance, as essential nutrients are withdrawn from the ecosystem. This is particularly critical in the case of K, as the spruces at the Höglwald site have already shown an insufficient supply of this nutrient element for several years of the study (Huber et al., 2006).

**FIGURE 5:**
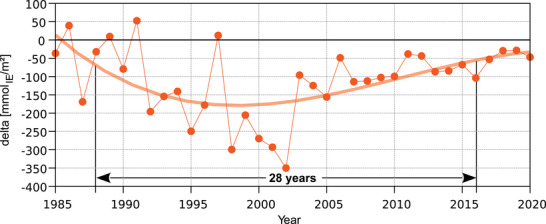
Change in S storage in the topsoil calculated as the difference between SO_4_^2−^ input to the soil by throughfall and SO_4_^2−^ output with seepage water at a depth of 40 cm at the Norway spruce forest Höglwald (Bavaria, Germany); the polynomial shown (degree 3) serves to better visualize the development over time and to derive the time span of marked S‐remobilization (delta S < −50 mmol_IE_ m^−2^).

During the periods of high S input, which according to Figure 1 was up to about 1988, sulfate was stored in the soil by sorption, as precipitate (e.g., Jurbanite) or as organic S (Alewell, 1995, 2001). This stored sulfate can then be released again in times of lower S inputs, contributing to increased S availability and also SO_4_^2−^ discharge for a certain period (Prechtel et al., 2001). Figure 5 shows an estimation for the development of S storage in the topsoil (to a depth of 40 cm) calculated from the SO_4_^2−^ input with throughfall and the SO_4_^2−^ output with the seepage water. The relatively small net S incorporation of trees at the Höglwald (5.5 mmol_IE_ m^−2^ year^−1^) can be neglected as well as S input by litter fall, which in the long term is compensated by an S uptake of a similar amount. Looking at the highly scattering data points, it can be seen that at the beginning of the time series, the input into the soil and the output from the soil were of a similar order of magnitude. The highest loss of sulfate from the topsoil, as indicated by a negative balance, could be observed in the years 1992–2002. After 2002, sulfate loss continued in a much weaker and decreasing form. The third‐order polynomial fit of the delta values has a mean absolute error of 52.8 mmol_IE_ m^−2^, describing the average fluctuation around the trend line. Thus, it is reasonable to use a value of ±50 mmol_IE_ m^−2^ around the zero line as a “natural fluctuation zone” and to consider only values lower than − 50 mmol_IE_ m^−2^ as a real loss of sulfur. Evaluating the trend line using this threshold reveals that sulfur stored in the topsoil from the time of high inputs was substantially released as sulfate from 1988 to 2016, a period of 28 years.

### 3.4 Development of the proton load

The acidification of ecosystems occurs by direct proton input with precipitation, primarily caused by the emission of SO_2_ and nitrogen oxides and their conversion to acids in the atmosphere. In addition, transformations of nitrogen compounds in the soil significantly affect the acidity of the soil (Rehfuess, 1990). The most important reactions releasing protons are ammonium uptake by plants (NH_4_^+^ → N_org_ + **H^+^**) and nitrification (NH_4_^+^ + 2 O_2_ → NO_3_^−^ + H_2_O + 2 **H^+^**). Processes that consume protons are nitrate uptake by plants (NO_3_^−^ + **H^+^** → N_org_), ammonification (R‐NH_2_ + H_2_O + **H^+^** → NH_4_^+^ + R‐OH), and denitrification (4 NO_3_^−^ + 4 **H^+^** + 5/6 C_6_H_12_O_6_ → 2 N_2_ + 5 CO_2_ + 7 H_2_O). Based on these main processes, a relatively simple scheme for estimating proton transformations associated with nitrogen transformations can be derived by comparing NH_4_^+^‐ and NO_3_^−^‐ fluxes in throughfall and seepage:

One proton is consumed per nitrate transformed (uptake and denitrification).
One proton is consumed per ammonium formed (ammonification).
One proton is released for each ammonium converted (uptake or nitrification).
One additional proton is released for each nitrate formed from ammonium (nitrification).

In principle, S‐transformations also have an impact on the proton budget. As we only measured sulfate, we do not know anything about other S‐species and thus cannot estimate any proton changes coupled to S‐transformations. However, the soil at the Höglwald is permanently aerobic. In an aerobic soil, reduced S‐species are of very minor importance and, consequently so are oxidation processes coupled to reduced S. Thus, unlike N, where the oxidation of NH_4_^+^ to NO_3_^−^ is an important source of protons, in our case, S has no decisive influence on the proton load.

**FIGURE 6:**
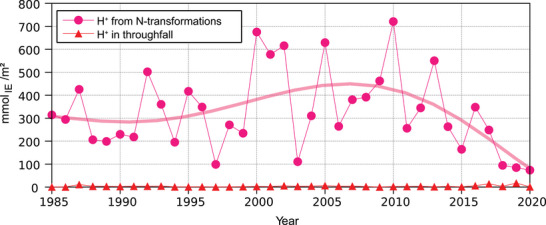
Temporal development of the proton load of the topsoil (humus + mineral soil to a depth of 40 cm) in the years 1985–2020 at the Norway spruce forest Höglwald (Bavaria, Germany); the polynomial shown (degree 5) serves to better visualize and interpret the temporal development.

As the input of ammonium by throughfall at the Höglwald site clearly exceeds the input of nitrate (Figure 3), the overall nitrogen transformations lead to acidification. Figure 6 shows the proton load of the topsoil, both by direct proton input with throughfall and by protons resulting from nitrogen transformations. With values between 0.4 and 17.7 mmol_IE_ m^−2^ year^−1^, the direct proton input with throughfall was almost negligible compared to the proton load resulting from nitrogen transformations (79.3–718.6 mmol_IE_ m^−2^ year^−1^). In the dry years 1997, 2003, and 2018–2020, the proton load resulting from nitrogen transformations was clearly lower than in other years and in total was strongly linked to nitrate discharge. The high linear correlation between proton load and nitrate discharge (*R* ^2^ = 0.974) highlights the importance of nitrification with respect to soil acidification in the Höglwald. There are many other processes in the upper soil that release or consume protons (exudation of organic acids by roots, uptake of cations or anions other than NH_4_^+^ and NO_3_^−^, redox‐processes of elements other than N, weathering of minerals, buffer reactions, etc.), but which cannot be estimated from the available data. However, the overall net effect of all these processes is indirectly also taken into account when calculating an input–output balance of known proton fluxes to and from the soil.

The percentage of protons that could be neutralized in the topsoil was estimated by comparing the proton input by throughfall and by nitrogen transformation with the proton output by seepage in 40‐cm depth. The proton load that could be buffered decreased from an average of 97.0% in the first 5 years of observation to 93.4% in the last 5 years of the measurement series. This indicates a slight but statistically significant trend of decreasing buffer capacity, making the stand more susceptible to further acidification.

## 4 SUMMARIZING DISCUSSION

There are two main pathways through which substances, in our case S and N, come into a forest ecosystem, wet and dry deposition. Wet deposition is covered by measuring bulk deposition. Dry deposition cannot be measured directly; however, it is indirectly part of throughfall. According to Adriaenssens et al. (2012), for S, there are only little exchange processes with the forest canopy. Therefore, the difference in fluxes between bulk deposition and throughfall can be attributed to dry deposition. In the case of N, however, the processes in the canopy are much more complicated. Trees can take up inorganic N (NH_4_^+^ and NO_3_^−^) in their canopy (Adriaenssens et al., 2012), as well as leach substantial amounts of organic‐bound N from their canopy (Matzner, 1988). Thus, for N, the difference of inorganic N between bulk deposition and throughfall is the net effect of all the aforementioned processes. However, the Höglwald was a nitrogen saturated ecosystem throughout the entire observation period, as evidenced by continuous nitrate output. Furthermore, there were only marginal changes in stand structure. Thus, for N, changes in the difference between bulk deposition and throughfall also can be attributed mainly to changes in dry deposition, as the contribution of all other aforementioned processes should have remained relatively constant.

The output by seepage at a depth of 40 cm is the result of all input fluxes to the soil surface (throughfall, litter fall) as well as all transformations and processes that occur in humus and mineral soil down to 40 cm, for example, mineralization, adsorption, desorption, weathering, plant uptake. However, most of these transformations and processes cannot be directly measured. Although the ecosystem, especially the soil, is something like a “black box” with respect to many processes, the resulting net effect of all unknown or unmeasurable processes is also represented in seepage chemistry.

Norway spruce is known to have a very shallow root system, which develops sinker roots close to the stem (Köstler et al., 1968). At the Höglwald, sinker roots are found up to a distance of 1.25 m from the center of the stem and can reach a depth of about 2 m. Thus, only about 30% of the stand area is accessed by deep roots. The remaining 70% is characterized by shallow rooting in the humus and upper mineral soil, with 95% of the fine root biomass found at depths down to 20 cm. Below a soil depth of 40 cm, virtually, no fine roots are present (Kreutzer et al., 1991). As the suction cups were installed at a depth of 40 cm in the area between the stems, the calculated fluxes are a good estimation for the output of N and S of the stand toward groundwater. The area close to the stems is very important for the trees, as here they gain stability through sinker roots giving access to deeper soil layers and thus providing additional nutrients and water. However, as estimated by Pröbstle and Kreutzer (1991), only about 11% of the total transpiration of the stand is coming from soil deeper than 40 cm in the close‐to‐stem area. Therefore, knowledge gained from the 70% of the area can be extrapolated to the entire area without too big an inaccuracy. The effect of the deeper rooting close to the stem with respect to nutrients is detected in an indirect way, as the nutrients taken up from deeper layers are spread also to the area between the stems by litterfall and leaching.

The Höglwald is a relatively small forest in an environment dominated by agriculture. Therefore, it is more influenced by agricultural emissions and thus by reduced nitrogen than forests located in larger forest landscapes (Göttlein and Kreutzer, 1991). The results from the Höglwald are therefore characteristic for a forest ecosystem that has been saturated with nitrogen for a long time, dominated by the input of reduced nitrogen. As nitrification, that is the conversion of ammonium to nitrate in the soil, is a major source of protons, the input of reduced nitrogen is a main driver of soil acidification. Thus, the current dominance of reduced nitrogen in emissions represents a burden for the forest ecosystem. At the Höglwald site, which has been exposed to high levels of reduced nitrogen for decades, the resulting relatively high proton load has already led to a small, but significant reduction in the ability of the topsoil to buffer protons. This means that the ecosystem will become increasingly sensitive to the continued input of nitrogen and the resulting proton load.

For ammonium and especially sulfate, it is striking that the difference in input between bulk precipitation and throughfall decreased significantly from 1985 to 2020. As these ions generally are not leached out of the canopy (Adriaenssens et al., 2012), this decrease is mainly due to a reduction in dry deposition, that is, the deposition of these two ions by aerosols, dust, and gases decreased significantly during the observation period. In the case of ammonium, this is a consequence of the structural change in agriculture, in the course of which the number of farms in the administrative district of Swabia has decreased by over 60% (1986: 36,634 farms [Bayerisches Landesamt f. Statistik, 1987]; 2020: 13,911 farms [Bayerisches Landesamt f. Statistik, 2022]). Consequently, livestock farming and thus ammonia emissions from stables and liquid manure spreading have also decreased significantly in this region. The decrease for cattle was about 40%, while the decrease for pigs was much lower, at about 6% (1986: 1,069,900 cattle, 642,540 pigs (Bayerisches Landesamt f. Statistik, 1987) equal to 1,172,706 livestock units; 2020: 605,700 cattle, 512,840 pigs (Bayerisches Landesamt f. Statistik, 2022) equal to 687,754 livestock units). The decrease of NH_4_^+^ in throughfall (49%) at the Höglwald and the decrease of livestock units (41%) in the entire district of Swabia are similar, indicating that, as expected, livestock farming is the primary driver of loading the ecosystem with reduced nitrogen.

In the case of sulfur, the reduction in deposition is due to the consistent reduction in emissions through the use of sulfur‐free fuels and the desulfurization of flue gases from large power plants (Umweltbundesamt, 2005). In contrast, there has been no reduction in dry deposition for nitrate. Nitrogen oxides are produced by any combustion process with a temperature above about 1000°C through the oxidation of atmospheric nitrogen (Bundesverband der Deutschen Heizungsindustrie, 2020), so they are emitted by all heating systems and also by combustion engines not having catalytic nitrogen reduction.

Through biomass analyses and stand increment data, for the Norway spruce stand at the Höglwald, an annual requirement of 9.7 kg ha^−1^ year^−1^ of nitrogen and 0.9 kg ha^−1^ year^−1^ of sulfur can be derived for the stand age of 95–105 years. In 1985, the deposition of nitrogen with throughfall was 4.7 times higher than the demand of the stand, and the sulfur deposition was 59.5 times higher. By 2020, the nitrogen surplus had decreased to a factor of 1.7 and the sulfur surplus to a factor of 1.4. In some forested areas, the drastic reduction in sulfur inputs has already led to sulfur deficiency (Göttlein and Mellert, 2018; Göttlein et al., 2020). At the Höglwald site, it is also possible that the sulfur supply of the forest stand will become critical in the future if further decreasing inputs cannot fully cover the needs of the stand and the sulfur quantities stored in the soil from periods of high inputs become mobilized and lost (see Figure 5). This is illustrated by comparing the input/output balance with the sulfur reserves in the topsoil (humus + mineral soil 0–40 cm deep) at the beginning of the study. According to Fischer (1986), these reserves were 2281.3 mmol m^−2^ (equivalent to 731.5 kg ha^−1^). In the humus, sulfur was stored exclusively in organically bound forms, while in the mineral soil, the organically bound fraction decreased from 89% at a depth of 0–5 cm to 36% at a depth of 30–40 cm. Summing the change in topsoil storage from 1985 to 2020 by subtracting the output flux of sulfate from the input fluxes, a value of −2012.3 mmol m^−2^ is obtained. Thus, in 2020, the topsoil contained only about 11% of the sulfur reserves present in 1985. Even though the complete discharge of accumulated sulfur from the entire rooting zone will take some time, sulfur is an element that can no longer be neglected when considering the sustainability of forest nutrient management. Nowadays, it matters how much sulfur is removed from the ecosystem by harvesting. As reported by Jonard et al. (2015), there is a more or less general trend of decreasing nutrient concentrations in European tree species, with a special emphasis on phosphorus. Therefore, not only with respect to S, an element that is shifting from excess to deficit (Göttlein and Mellert, 2018), material rich in nutrients, such as leaves/needles, twigs, branches, and as far as possible also bark, should be left in the forest and not removed by harvesting.

There are two Norway spruce forests in Germany, where element inputs and outputs have been measured continuously for even longer than at the Höglwald site. One is a stand in the Solling (since 1969; Ellenberg et al., 1986), and the other a stand at Lange Bramke in the Harz (since 1981; Hauhs, 1989). In these two forests, the input of sulfate also decreased drastically during the observation period. As in the Höglwald, the difference between stand and field precipitation almost disappeared at both sites, at the Lange Bramke from around 2000 on, at the Solling from around 2020 (Scheler, 2022). Unfortunately, no runoff data are published for the two sites for the period up to 2020, so a real comparison with the Höglwald time series is not possible. For the Lange Bramke site, an evaluation of sulfate discharges up to 2013 can be found in Müller et al. (2015), which shows that the soil (down to 80‐cm depth) stored sulfate before 1992 and became a source of sulfate after this time. Also, at the spruce forest in Solling an evaluation of annual SO_4_^2−^ fluxes for the years 1975 to 2011 shows, that since 1991 output at a soil depth of 90 cm is higher than the input (Meesenburg et al., 2016). The year from which soil acts as source for SO_4_^2−^ at Lange Bramke and Solling is similar to the Höglwald, where the sulfate stored in the soil from times of high inputs was also increasingly mobilized since 1988.

## 5 CONCLUSION

The changes in emissions since the beginning of industrialization and the drastic shift in the S/N ratio during the measurement period from 1985 to 2020 have changed the quantity and quality of inputs into the forests to such an extent that we can speak of a change in the chemical climate in Central Europe. This is particularly true for sulfur, as inputs into forest ecosystems have decreased from a problematic surplus to amounts close to or even lower than the sulfur requirements of forests in many regions. This means that although there is a delay of about 30 years, future forest management should aim to minimize sulfur removals related to harvesting, as has long been known for other macronutrients such as phosphorus. Although nitrogen inputs have decreased in recent decades, they still exceed the nitrogen requirements of the forest ecosystem with the known negative consequences such as a shift toward nitrophilic soil vegetation and high nitrate leaching with the seepage water. At sites where reduced nitrogen is dominating N‐input, such as the Höglwald, nitrification is an important driver of soil acidification. Changes in the nutrient balance of forest ecosystems can only be detected and reasonably interpreted through long‐term observations. Therefore, long time series have a value “in themselves”, which is why securing long‐term funding for maintaining measurement series is a political task, for example, as part of the Level II program.

## AUTHOR CONTRIBUTIONS

**A. Göttlein**: Conceptualization; visualization; writing—original draft; writing—review and editing. **W. Weis**: Data curation; software; writing—original draft. **S. Raspe**: Data curation; writing—original draft.

## CONFLICT OF INTEREST STATEMENT

The authors declare no conflict of interest.
